# Draft Genome Sequences of Four Pseudomonas aeruginosa Clinical Strains with Various Biofilm Phenotypes

**DOI:** 10.1128/MRA.01286-19

**Published:** 2020-01-02

**Authors:** Amine M. Boukerb, Marjolaine Simon, Erwan Pernet, Albane Jouault, Emilie Portier, Elise Persyn, Emeline Bouffartigues, Alexis Bazire, Sylvie Chevalier, Marc G. J. Feuilloley, Olivier Lesouhaitier, Jocelyne Caillon, Alain Dufour

**Affiliations:** a Laboratoire de Microbiologie Signaux et Micro-Environnement, LMSM EA4312, Université de Rouen-Normandie, Normandie Université, Evreux, France; b Université de Bretagne-Sud, EA 3884, LBCM, IUEM, Lorient, France; c Université de Nantes, Faculté de Médecine, EA3826 Thérapeutiques Cliniques et Expérimentales des Infections, Nantes, France; University of Maryland School of Medicine

## Abstract

Biofilms produced by Pseudomonas aeruginosa present a serious threat to cystic fibrosis patients. Here, we report the draft genome sequences of four cystic fibrosis isolates displaying various mucoid and biofilm phenotypes. The estimated average genome size was about 6,255,986 ± 50,202 bp with a mean G+C content of 66.52 ± 0.06%.

## ANNOUNCEMENT

The lungs of cystic fibrosis (CF) patients are prone to infections by Pseudomonas aeruginosa (1). CF lungs are generally colonized by nonmucoid P. aeruginosa strains forming biofilms, and chronic infections occur upon the emergence of mucoid strains overproducing alginate (2). Their biofilms are highly resistant to antibiotics and immune mediators and lead to pulmonary decline (2, 3).

P. aeruginosa strains were isolated from sputum samples of adult CF patients suffering from chronic infection and followed at the Centre Hospitalier Universitaire, Nantes, France. Since these sputum samples were used only to isolate bacteria but not to work on human cells or on human DNA, French law (Décret no 2016-1537, 16 November 2016) does not require that the study be reviewed and approved by an institutional ethics committee or that the participants provide their written or verbal informed consent. Bacteria from sputum samples were grown on blood agar medium at 37°C and identified as P. aeruginosa using matrix-assisted laser desorption ionization–time of flight mass spectrometry (MALDI-TOF MS [Vitek; bioMérieux, Marcy-l’Étoile, France]). A single isolate from each patient was used. Based mainly on their biofilm structure and mucoid phenotype, the isolates MUC-N1, MUC-N2, MUC-P4, and MUC-P5 were selected to constitute a strain panel for testing antibiofilm compounds (M. Simon, E. Pernet, A. Jouault, E. Portier, A. M. Boukerb, S. Pineau, J. Vieillard, E. Bouffartigues, C. Poc-Duclairoir, M. G. J. Feuilloley, O. Lesouhaitier, J. Caillon, S. Chevalier, A. Bazire, and A. Dufour, submitted for publication), which prompted us to sequence their genomes.

Each strain was grown at 37°C in liquid LB medium inoculated with a single colony picked from an LB agar plate, and its genomic DNA was extracted using a GeneJet genomic DNA purification kit (Thermo Fisher Scientific, France) following the manufacturer’s recommendations and assessed using the double-stranded DNA (dsDNA) high-sensitivity kit on a Qubit fluorometer (Thermo Fisher Scientific, USA) and 1% agarose gel electrophoresis. Sequencing libraries were prepared using the Illumina Nextera XT DNA library prep kit following the manufacturer’s protocol. Pooled libraries were sequenced on a MiSeq instrument (LMSM Genomics platform, Rouen Normandy University, Evreux, France) with dual-index paired-end reads using the MiSeq reagent kit v.3 (2 × 250 bp). Default parameters were used for all software unless otherwise noted. The reads were trimmed using Trimmomatic v.0.36 (4), and their quality was checked with MultiQC v.1.6 (5). *De novo* assembly was performed with Unicycler v.0.4.7 (6), and assembly metrics were calculated using QUAST v.5.0.0 (7). Prokka v.1.14.0 (8) was used for structural gene prediction and functional annotation. Multilocus sequence typing (MLST), the resistome, and the virulome were assessed using the “reads to report” Nullarbor pipeline v.2.0.20181010 (9), with sequence identity and coverage thresholds fixed at 70% and 90%, respectively. Prophage regions were identified through the integrated search and annotation tool PHASTER (10).

The average total size of the draft genomes was 6,255,986 ± 50,202 bp, arranged into 100 ± 8 contigs (Table 1). The genome sequence data were at an average coverage of 64 ± 3× with a mean *N*_50_ value of 185,027 ± 24,962 bp and a mean G+C content of 66.52 ± 0.06%. These genomes contain an average of 5,706 ± 90 coding sequences (CDS), genes for 3 rRNAs, 65 ± 3 tRNAs, and 1 transfer-messenger RNA (tmRNA). MLST typing identified MUC-N1 and MUC-N2 with sequence type 549 (ST549), MUC-P4 with ST675, and MUC-P5 with ST649, all of which clustered within the PAO1 clade. Major virulence factors involved in human pathogenicity were identified, including the *exoS*, *exoT*, and *exoY* genes. The four strains shared the following antibiotic resistance genes: *aph(3′)-IIb* (aminoglycosides), *bla*_PDC-55_ (beta-lactam antibiotics), *catB7* (phenicols), and *fosA* (phosphonic acids). Three additional beta-lactam resistance genes were found, *bla*_OXA-50_ in MUC-N1 and MUC-N2, *bla*_OXA-395_ in MUC-P4, and *bla*_OXA-396_ in MUC-P5. Ten prophage loci were predicted (eight different prophages), five of which are complete (Table 1). MUC-P5 contains six prophages (three are complete), whereas the other strains contain one or two prophages. Altogether, the genetic features of these four strains are of interest, especially in the perspective of using them as a panel to investigate antibiofilm molecules.

**TABLE 1:** Overview of the draft genome assemblies of the four P. aeruginosa strains

Parameter	Data for strain:			
	MUC-N1	MUC-N2	MUC-P4	MUC-P5
GenBank accession no.	WBZH00000000	WBZG00000000	WBZF00000000	WBZE00000000
SRA accession no.	SRR10230517	SRR10230516	SRR10230515	SRR10230514
No. of total reads	1,617,598	1,508,174	1,658,332	1,669,232
Mean coverage (×)	65	61	67	66
G+C content (%)	66.58	66.58	66.50	66.46
*N*_50_ (bp)	185,163	162,332	160,065	209,989
No. of contigs	105	98	108	92
Genome size (bp)	6,206,374	6,205,784	6,209,920	6,306,189
No. of CDS	5,642	5,634	5,616	5,795
No. of tRNAs	67	67	68	62
No. of rRNAs	3	3	3	3
Prophage loci^*a*^	Pseudo YMC11/02/R656 (NC028657)	Pseudo YMC11/02/R656 (NC028657)	Pseudo YMC11/02/R656* (NC028657); Pseudo phiCTX* (NC003278)	Pseudo B3 (NC006548); Escher vB EcoM ECO1230 10 (NC027995); Vibrio VP882 (NC009016); Shigel SfIV* (NC022749); Burkho BcepMu* (NC005882); Pseudo Pf1* (NC001331)

a Asterisks (*) indicate incomplete prophage loci.

### Data availability.

The draft sequences have been deposited at DDBJ/ENA/GenBank under the accession numbers cited in Table 1.
